# Comparison of drug regimens for recurrent or metastatic cervical cancer: a systematic review and network meta-analysis

**DOI:** 10.3389/fimmu.2026.1775409

**Published:** 2026-02-25

**Authors:** Jin Zhou, Wentao Ye, Stéphanie Nirina Ranarisoa, Lei Tian

**Affiliations:** International Pharmaceutical Business School, China Pharmaceutical University, Nanjing, Jiangsu, China

**Keywords:** cervical cancer, drug therapy, immunecheckpoint inhibitors, metastatic, network meta-analysis, recurrent

## Abstract

**Background:**

Cervical cancer is one of the most common cancers among women worldwide. For patients with recurrent or metastatic cervical cancer (R/MCC) after surgery or radiotherapy, drug therapy is the primary treatment modality. Currently, head-to-head comparison studies of different immune checkpoint inhibitors (ICI) combination regimens are lacking in clinical practice. This study aims to provide an indirect comparison of the relative efficacy of various drug regimens (including chemotherapy, targeted therapy, and immunotherapy) for R/MCC patients through a systematic review and network meta-analysis (NMA).

**Method:**

The study adhered to the Preferred Reporting Items for Systematic Reviews and Meta-analyses (PRISMA) reporting guidelines and systematically searched databases including PubMed, Web of Science, Embase and the Cochrane Library for randomized controlled trials (RCTs) comparing drug treatment regimens. The primary efficacy endpoint was overall survival (OS). Progression-free survival (PFS) was analyzed as a **s**econdary endpoint to provide additional evidence of clinical activity. We conducted the NMA using a Bayesian random-effects model, estimated the ranking of each treatment regimen via the Surface Under the Cumulative Ranking Curve (SUCRA), and performed a Frequentist NMA as a sensitivity analysis.

**Result:**

A total of 15 RCTs involving 4,588 R/MCC patients were included. The NMA results for OS showed that ICI combination regimens (with or without bevacizumab) provided a significant benefit compared to backbone chemotherapy. Specifically, the regimen of pembrolizumab plus chemotherapy and bevacizumab showed the greatest potential for OS benefit (Frequentist HR: 0.45, 95%CI: 0.30–0.67 vs. cisplatin plus paclitaxel), ranking first by SUCRA (87%). Among traditional chemotherapy regimens, only the cisplatin plus paclitaxel regimen was significantly superior to single-agent cisplatin (Frequentist HR: 0.74, 95%CI: 0.59–0.93). The NMA results for PFS indicated that the cadonilimab plus chemotherapy regimen was the most outstanding (Frequentist HR: 0.46, 95%CI: 0.32–0.66 vs. cisplatin plus paclitaxel), ranking first by SUCRA (90%). The rankings of the treatment regimens were consistent across both Bayesian and Frequentist, suggesting strong robustness of the results.

**Conclusion:**

ICI combination regimens (with or without bevacizumab) are likely the optimal choice for treating R/MCC patients. Pembrolizumab plus chemotherapy and bevacizumab is most likely to yield the OS benefit, and cisplatin plus paclitaxel remains the best backbone chemotherapy regimen for R/MCC. This study provides comprehensive indirect comparison evidence for clinicians in selecting R/MCC treatment strategies.

**Systematic Review Registration:**

https://www.crd.york.ac.uk/PROSPERO/view/CRD420251180897, identifier CRD42024604107.

## 1 Introduction

Cervical cancer is primarily caused by infection with human papillomavirus (HPV) and remains the fourth most common cancer among women globally (1). In 2022, there were approximately 660,000 new cases of cervical cancer worldwide, with an average incidence of 74.9 per 100,000 women, resulting in a significant economic burden, particularly in high-incidence, low-income regions (2). To address this public health challenge, preventive measures such as HPV vaccination and early screening have been implemented in various countries and regions. However, the treatment of cervical cancer continues to face difficulties, especially in patients with recurrent or metastatic disease (R/MCC) following surgery or radiotherapy, where existing treatment modalities offer limited efficacy, making management particularly challenging (3).

For the majority of R/MCC patients, drug therapy is the mainstay of treatment, including chemotherapy, targeted therapy, and immunotherapy. Currently, first-line treatment guidelines recommend the use of immune checkpoint inhibitors (ICIs) combined with chemotherapy, with or without bevacizumab, depending on the clinical context (4). Chemotherapy regimens typically involve cisplatin or carboplatin combined with paclitaxel, and non-platinum regimens are also used clinically. The application of targeted therapies and ICIs has significantly improved the treatment outcomes for R/MCC. Nevertheless, head-to-head comparison studies of different ICI combination regimens are still lacking.

The emergence of multiple therapeutic options offers hope to patients while simultaneously imposing higher demands on clinicians' decision-making capabilities, who must comprehensively weigh treatment costs, efficacy and suitable patient populations. Studies have confirmed that ICI combined with chemotherapy is superior to chemotherapy alone (5), but the comparative efficacy of different drug regimens for R/MCC remains insufficient, especially the comparison among different ICI regimens. Therefore, it is necessary to conduct a network meta-analysis (NMA) to provide indirect comparison evidence on the relative efficacy of various drug regimens, thereby offering a robust basis for clinical decision-makers.

## 2 Materials and methods

### 2.1 Literature search strategy

The study was conducted in accordance with the Preferred Reporting Items for Systematic Reviews and Meta-analyses (PRISMA) reporting guidelines. The protocol was registered in the Prospective Register of Systematic Reviews (ID: CRD 420251180897, https://www.crd.york.ac.uk/PROSPERO/view/CRD420251180897). We systematically searched the PubMed, Web of Science, Embase and Cochrane Library databases from inception to November 10, 2025. To minimize the risk of bias, we supplemented our search by examining the ClinicalTrials.gov registry and manually searched conference abstracts from the American Society of Clinical Oncology (ASCO), the European Society for Medical Oncology (ESMO), and the Society of Gynecologic Oncology (SGO) to identify unpublished gray literature. The comprehensive search keywords included (Cervical Cancer) AND (Drug Therapy) AND (Clinical Trial). The specific search strategy is detailed in the **Supplementary Material 1**.

### 2.2 Inclusion and exclusion criteria

This study included patients with histologically confirmed R/MCC, focusing on randomized controlled trials (RCTs) that compared drug treatment regimens. Trials had to compare any drug regimen (including chemotherapy, targeted therapy, or immunotherapy) against a concurrent control arm (such as standard treatment or placebo) and must report the hazard ratios (HR) for overall survival (OS) and/or progression-free survival (PFS), or provide sufficient data to calculate these HR. Studies with non-randomized designs, those involving non-metastatic or non-recurrent cervical cancer patients, and those where the intervention was non-drug therapy or lacked a concurrent control group were excluded. Furthermore, Phase I clinical trials, non-clinical studies, duplicate publications, and studies for which the full text could not be obtained, which did not report relevant survival outcome HR and from which relevant data could not be extracted, were also excluded.

### 2.3 Literature screening and data extraction

Literature screening was performed independently by two researchers, followed by cross-validation. Any disagreement was resolved by consulting a third party. Initial screening was performed by reading titles and abstracts, and further screening involved reading the full text in conjunction with the inclusion and exclusion criteria. Data were extracted by two researchers. Extracted items mainly included: first author and publication year, characteristics of the included population, sample size, interventions in the treatment and control groups, median OS and PFS, and the HR and confidence intervals (CIs) for each intervention. Additionally, if a study did not report the HR but provided the corresponding Kaplan-Meier survival curves, we used the WebPlotDigitizer software to extract individual patient data, and utilized the IPDfromKM R package developed by Liu et al. to calculate the HR and its standard deviation (6).

### 2.4 Risk of bias assessment

Two reviewers independently assessed the risk of bias using the Cochrane Risk of Bias Tool 2 (RoB 2.0). Each study was rated as low risk, high risk, or some concerns, with disagreements resolved through discussion. RoB 2.0 covers five domains: randomization process, deviations from intended interventions, missing outcome data, measurement of the outcome, and selection of the reported result (7). The robvis R package was used to visualize the assessment results (8).

### 2.5 Data processing and statistical analysis

Given that the NMA model requires good connectivity and needs to avoid over-complication, we merged interventions with similar therapeutic effects: (1) Regimens combining placebo with other drugs were considered the same node as the single drug regimen, as placebo does not affect efficacy; (2) Cisplatin or carboplatin plus paclitaxel regimens were simplified to cisplatin plus paclitaxel. For instance, cadonilimab plus cisplatin or carboplatin + paclitaxel was simplified to cadonilimab plus cisplatin + paclitaxel. This decision was based on two considerations: First, in trials comparing platinum-doublet chemotherapy with other therapies, the specific platinum agent was chosen based on clinical practice, and the randomization process itself ensured baseline comparability between groups, thus balancing the potential impact of the choice of platinum agent. Second, a head-to-head comparison study (JGOG-0505) in R/MCC had already demonstrated that carboplatin plus paclitaxel was non-inferior to cisplatin plus paclitaxel regarding OS and PFS (9).

In addition, the study selected the HR as the effect size and conducted NMA using both Bayesian and Frequentist approaches. Specifically, Bayesian network meta-analyses were performed using the gemtc package in R, applying a random-effects model under the consistency assumption, with four Markov chain Monte Carlo (MCMC) chains run. For the Bayesian inference, non-informative (vague) priors were specified to ensure that results were data-driven: relative treatment effects ( ${\text{d}}_{\text{ik}}$) were assigned a normal distribution $\text{N}\left(0,100^{2}\right)$, while the study-to-study standard deviation τ) was assigned a uniform distribution U(0,om.scale). Four Markov chain Monte Carlo (MCMC) chains were run, each undergoing 5,000 burn-in iterations followed by 50000 formal iterations with a thinning interval of 5, yielding 40000 effective posterior samples.

Network inconsistency was assessed via both local and global approaches. Local inconsistency was evaluated using the node-splitting method, while global inconsistency was assessed by fitting an unrelated mean effects (UME) model (the design-by-treatment interaction test). Model fit and consistency were appraised by comparing the Deviance Information Criterion (DIC) values between the consistency and UME models.

Heterogeneity was quantified not only by the ${\text{I}}^{2}$ and Cochran's Q statistics but also by reporting the posterior estimates of the between-study variance ( ${\text{τ}}^{2}$) derived from the random-effects model. To ensure the robustness of the results, a prior sensitivity analysis was conducted by comparing the primary model with an alternative specification using a half-normal prior ( τ∼HN(0,1)). Finally, the SUCRA method was used for treatment ranking, and a Frequentist analysis using the netmeta package was performed for cross-validation.

## 3 Results

### 3.1 Literature screening and study characteristics

**Figure 1:**
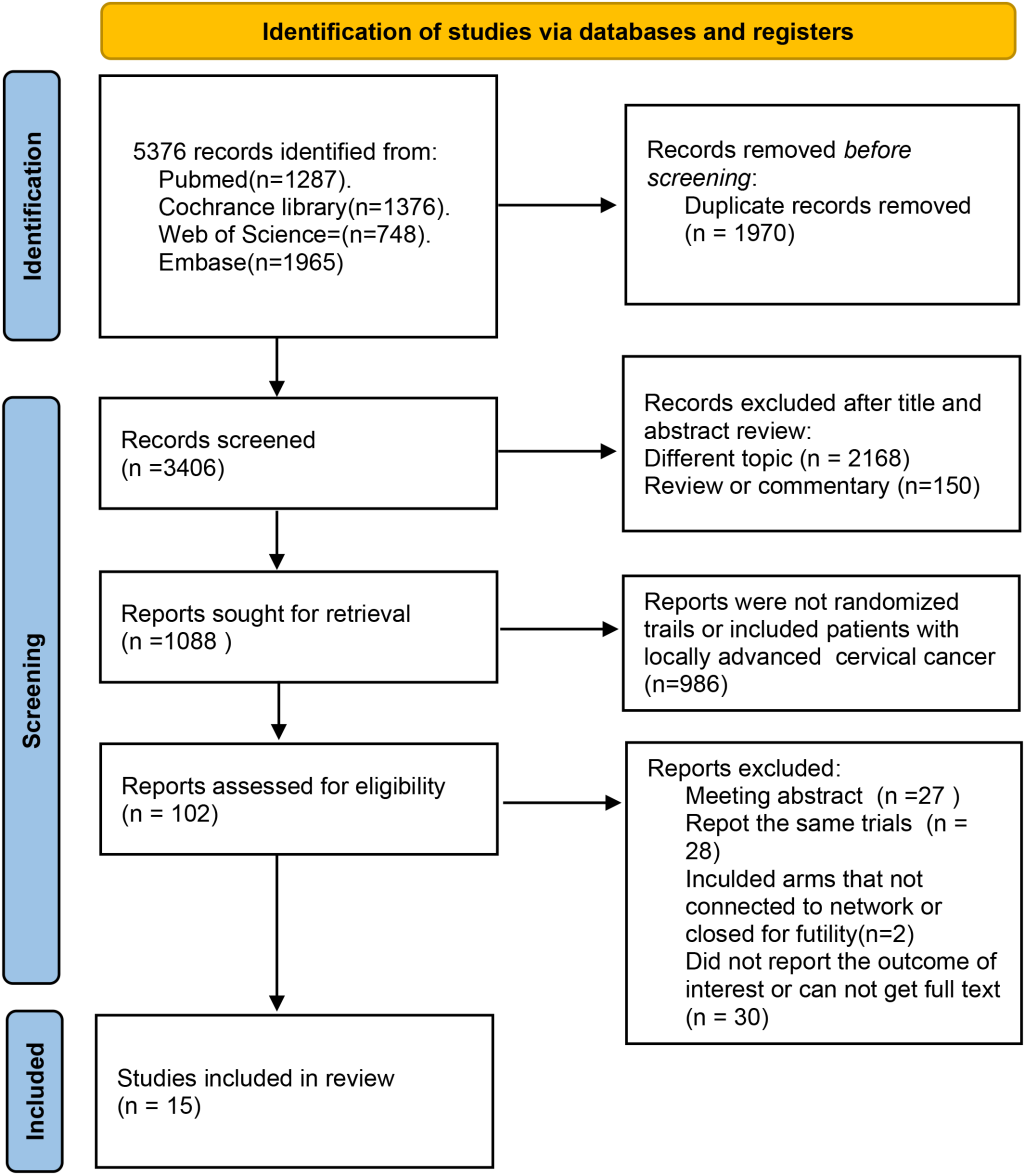
PRISMA flow diagram.

A total of 5376 records were retrieved through the established search strategy. After removing duplicates, 3406 records remained for screening by title and abstract. Following a careful full-text review of the remaining 102 articles, and based on the inclusion and exclusion criteria, 15 RCTs were finally included. The detailed screening process is shown in **Figure 1**.

The 15 included studies comprised 4 phase II clinical trials and 11 phase III clinical trials, involving a total of 4,588 patients with R/MCC. Detailed characteristics of the included studies are presented in **Table 1**. 11 studies were rated as having a low risk of bias, and 4 studies were rated as having some concerns. Across the included clinical trials, the patient populations were predominantly composed of squamous cell carcinoma cases (accounting for 70% to 85%), and all patients had stage IVB R/MCC. Moreover, the reported median age ranges were highly consistent, falling between 46 and 55 years. This high degree of homogeneity in baseline characteristics provides a solid foundation for the transitivity assumption required in the subsequent network meta-analysis. The detailed risk of bias and clinical characteristics for the included studies were provided in the **Supplementary Material 1**.

**Table 1:** Characteristics of included studies.

Author,year	Trial name / registration ID	N	Histology(%)	Stage(%)	Exp arm	Ctr arm	Age(Exp vs Ctrl median)	OS( Exp vs ctr)	PFS( Exp vs Ctr)	Risk of bias (OS)
Omura,1997 (10),	GOG-110 / NA	438	SCC(100)	IVB,recurrent,persistent(100)	CIS_IFO	CIS	46.3 vs 47.3	8.3vs 8	4.6 vs 3.2	Some
					CIS_MIT	CIS	48.8 vs 47.3	7.3 vs8	3.3 vs 3.2	
Moore,2004 (11),	GOG-169 / NA	264	SCC(100)	IVB,recurrent,persistent(100)	CIS_PTX	CIS	48.5 vs 46	9.7 vs 8.8	4.8 vs 2.8	Some
Long,2005 (12),	GOG-179 / NA	294	SCC(85);nSCC(15)	IVB(12);Persistent(10);Recurrent(78)	CIS_TOP	CIS	46 vs 48	9.4 vs 6.5	4.6 vs 2.9	Low
Monk,2009 (13),	GOG-204 / NA	434	SCC(77);nSCC(13)	IVB(17),persistence(12),recurrent(71)	CIS_VIN	CIS_PTX	49 vs 50	9.99 vs 12.87	3.98 vs 5.82	Low
					CIS_GEM	CIS_PTX	45 vs 50	10.28 vs 12.87	4.70 vs 5.82	
					CIS_TOP	CIS_PTX	45 vs50	10.25 vs 12.87	4.57 vs 5.82	
Mountzios,2009 (14),	NA / NA	149	SCC(73);nSCC(27)	primary metastatic or recurrent(100)	CIS_IFO_PTX	CIS_IFO	50 vs 55	15.4 vs 13.2	7.9 vs 6.3	Some
Kitagawa,2015 (9),	JCOG-0505 / NCT00295789	253	SCC(83);nSCC(13)	IVB or persistent(20%),recurrence(80)	CAR_PTX	CIS_PTX	53 vs 53	18.3 vs 17.5	6.9 vs 6.2	Low
Symonds,2015 (15),	CIRCCa / ISRCTN23516549	69	SCC(69);nSCC(31)	relapse(13),metastases(30);both(57)	CAR_PTX_CED	CAR_PTX	44 vs 44	14.8 vs 13.6	8.1 vs 6.7	Low
Tewari,2017 (16),	GOG240 / NCT008030	452	SCC(69);nSCC(21)	IVB,Recurrent,Persistent,Metastatic(100)	CIS_PTX_BEV	CIS_PTX	/	17.5 vs 15.0	/	Low
					TOP_PTX_BEV	TOP_PTX	/	16.2 vs 12	/	
Aoki,2018 (17),	NA / NCT00770874	364	SCC(74);nSCC(26)	IVB(13),Recurrent(74),Persisten(13)	S1_CIS	CIS	55 vs 52.5	21.9 vs 19.5	7.3 vs 4.9	Low
Pignata,2019 (18),	MITO CERV-2 / NCT00997009	107	SCC(79);ADC(21)	advanced or recurrent(100)	CAR_PTX_CET	CAR_PTX	47 vs 52	17 vs 17.7	7.6 vs 5.2	Some
Vergote,2023 (19),	BGOG/ENGOT-cx1 / NCT 02 00 95 79	120	SCC(62);nSCC(38)	IVB or recurrent/persistent(100)	CAR_PTX_MIN	CAR_PTX	49.9 vs 48.7	21.7 vs 16.4	7.8 vs 5.8	Low
Gass,2024 (20),	AGO-Zervix-1 / NCT01405235	172	SCC,ADC(100)	Distant metastasis(74);Local recurrence(26)	TOP_PTX	CIS_TOP	50.4 vs 49	9.6 vs 12	4.4 vs 4.2	Low
Oaknin,2024 (21),	BEATcc / NCT03556839	410	SCC(78);nSCC(22)	IVB(22),Recurrent(73),Persistent(5)	ATE_CIS_PTX_BEV^*^	CIS_PTX_BEV^*^	51 vs 52.5	32.1 vs 22.8	13.7 vs 10.4	Low
Wu,2024 (22),	COMPASSION-16 / NCT04982237	445	SCC(83);nSCC(17)	IVB, persistent, recurrent, or metastatic (100)	CAD_CIS_PTX_BEV^*^	CIS_PTX_BEV^*^	/	Not reach	15.1 vs 11.5	Low
					CAD_CIS_PTX^*^	CIS_PTX^*^	/	28.2 vs. 15.1	11.7 vs 6.9	
Lorusso,2025 (23),	KEYNOTE-826/ NCT03635567	617	SCC(62);nSCC(38)	Metastatic(20),Persistent or recurrent (80)	PEM_CIS_PTX_BEV^*^	CIS_PTX_BEV^*^	51 vs 50	37.6 vs 22.5	15.2 vs 10.2	Low
					PEM_CIS_PTX^*^	CIS_PTX^*^	52.5 vs 50.0	17.1 vs 12.6	6.3 vs 6.2	

Exp, experimental; Ctr, control; SCC, squamous cell carcinoma; nSCC, non-squamous; cell carcinoma; ADC, adenocarcinoma; CIS, cisplatin; MIT, mitolactol; PTX, paclitaxel; TOP, topotecan; VIN, vinorelbine; GEM, gemcitabine; CAR, carboplatin; CED, cediranib; BEV, bevacizumab; S1, teysuno; CET, cetuximab; MIN, nintedanib; ATE, atezolizumab; CAD, cadonilimab; PEM, pembrolizumab; *: Regimens involving "cisplatin or carboplatin plus paclitaxel" were analyzed as "cisplatin plus paclitaxel (CIS_PTX)" to ensure network connectivity and reflect their clinical interchangeability as a backbone in first-line therapy.

### 3.2 Network meta-analysis of OS

**Figure 2:**
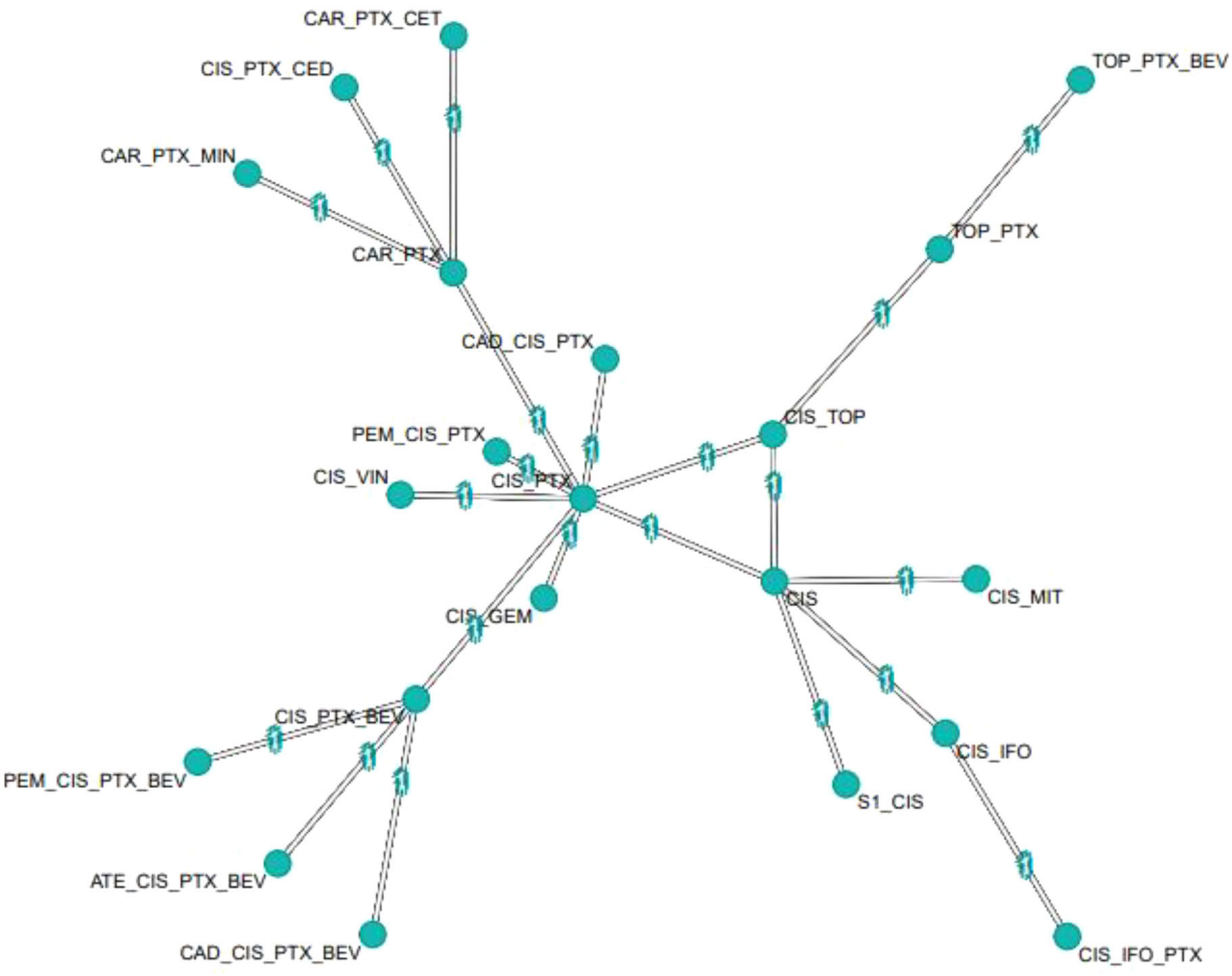
Network plot of different treatment regimens (OS). CIS, cisplatin; MIT, mitolactol; PTX, paclitaxel; TOP, topotecan; VIN, vinorelbine; GEM, gemcitabine; CAR, carboplatin; CED, cediranib; BEV, bevacizumab; S1, teysuno; CET, cetuximab; MIN, nintedanib; ATE, atezolizumab; CAD, cadonilimab; PEM, pembrolizumab.

All studies (9–23) were utilized for the NMA of OS, involving 21 interventions. The comparison relationships among these interventions are shown in **Figure 2**. Cisplatin plus paclitaxel served as the central node, directly comparing with various other treatment regimens. Simultaneously, the network map exhibited closed loops, allowing for the assessment of inconsistency.

**Figure 3:**
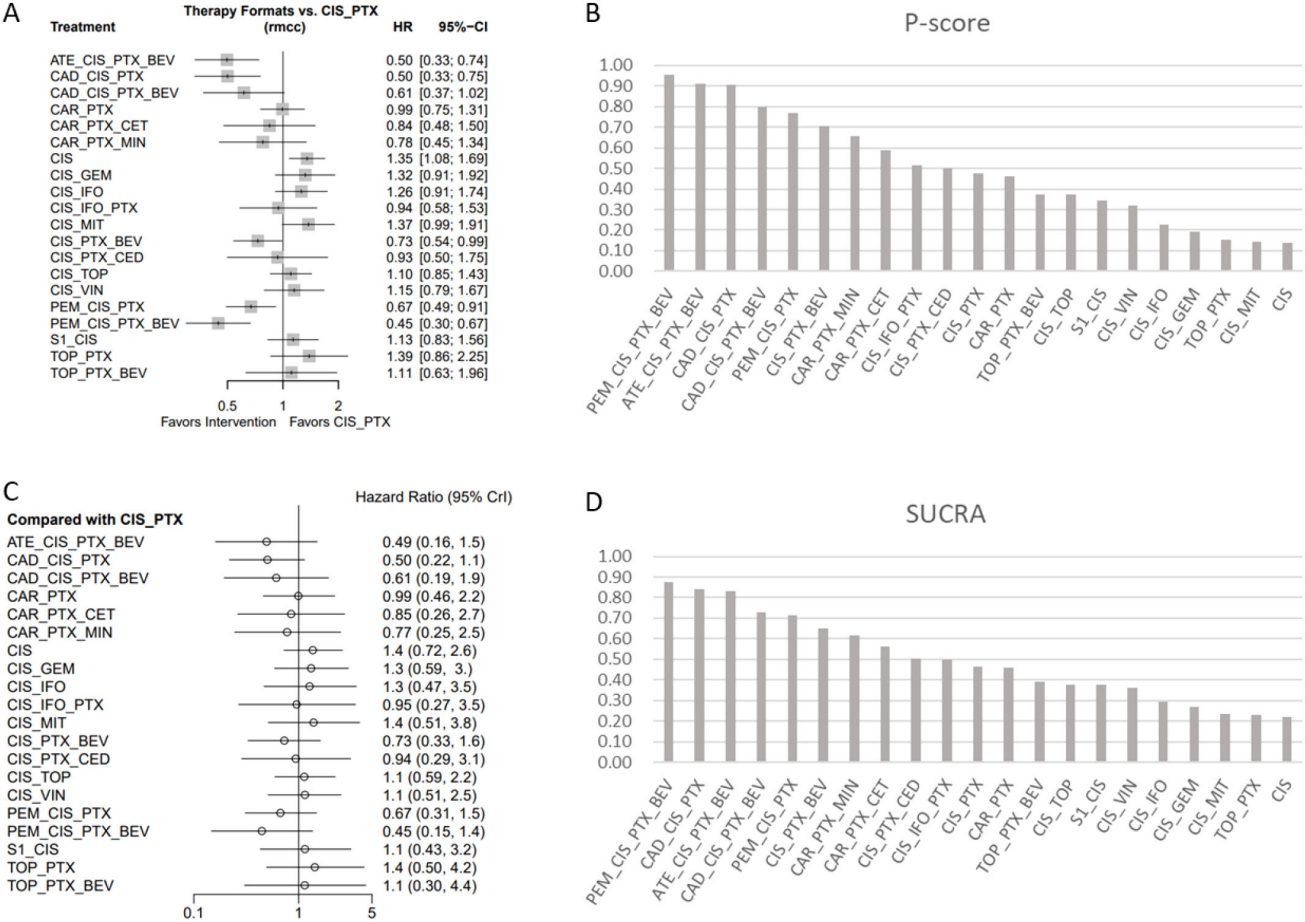
OS comparisons and ranking based on statistical methods. **(A, B)** show the comparison of different treatment regimens versus the cisplatin plus paclitaxel regimen for OS, and the probability ranking under the Frequentist method, respectively. **(C, D)** show the comparison of different treatment regimens versus the cisplatin plus paclitaxel regimen for OS, and the SUCRA ranking under the Bayesian method, respectively. CIS, cisplatin; MIT, mitolactol; PTX, paclitaxel; TOP, topotecan; VIN, vinorelbine; GEM, gemcitabine; CAR, carboplatin; CED, cediranib; BEV, bevacizumab; S1, teysuno; CET, cetuximab; MIN, nintedanib; ATE, atezolizumab; CAD, cadonilimab; PEM, pembrolizumab.

As shown in **Figure 3A**, multiple treatment regimens demonstrated a significant OS benefit compared to cisplatin plus paclitaxel under the Frequentist method. These primarily included immunotherapy regimens such as pembrolizumab plus chemotherapy (with or without bevacizumab), cadonilimab plus chemotherapy (with or without bevacizumab), and atezolizumab plus chemotherapy and bevacizumab. Among these, the regimen of pembrolizumab plus chemotherapy and bevacizumab showed the superior effect (HR: 0.45, 95%CI: 0.30–0.67), suggesting it may be the optimal treatment strategy for improving survival in this patient population. However, it is noteworthy that these differences were not significant under the Bayesian method (**Figure 3C**). This may be attributed to the Bayesian analysis providing a more complete quantification of uncertainty compared to the Frequentist method, particularly in handling random effects and model parameter uncertainty (24).

Concurrently, in the pairwise comparisons under the Frequentist framework (**Table 2**), single-agent cisplatin showed a higher risk compared to cisplatin plus paclitaxel (HR: 1.35, 95%CI: 1.08–1.69). Other doublet chemotherapy regimens (e.g., topotecan plus paclitaxel) did not show a benefit or a significant benefit compared to single-agent cisplatin. Notably, the carboplatin plus paclitaxel regimen showed a small and non-significant difference in OS benefit compared to the cisplatin plus paclitaxel regimen (HR: 0.99, 95%CI: 0.75–1.31). Detailed pairwise comparison results are available in the **Supplementary Material**. This suggests that the cisplatin plus paclitaxel regimen is likely the best chemotherapy backbone for R/MCC, with the carboplatin plus paclitaxel regimen showing minimal difference.

**Table 2:** Pairwise comparisons of the 11 interventions: HR (95%CI or 95% CrI).

ATE_CIS_PTX_BEV	1.01(0.26,3.9)	1.24(0.4,3.72)	2(0.52,7.39)	1.68(0.34,8.21)	1.57(0.33,7.34)	2.74(0.79,9.55)	2.66(0.68,10.22)	2.55(0.6,11.18)	1.91(0.38,10.27)	2.8(0.65,12.19)
0.99(0.56,1.76)	CAD_CIS_PTX	1.23(0.3,4.8)	1.99(0.63,5.98)	1.69(0.4,6.74)	1.56(0.38,6.08)	2.73(0.97,7.66)	2.65(0.84,8.23)	2.55(0.71,9.17)	1.91(0.44,8.73)	2.79(0.78,9.88)
0.81(0.50,1.31)	0.82(0.42,1.56)	CAD_CIS_PTX_BEV	1.6(0.42,6.3)	1.35(0.27,6.75)	1.26(0.26,6.26)	2.21(0.61,8.24)	2.14(0.56,8.64)	2.06(0.48,9.46)	1.55(0.29,8.63)	2.25(0.51,10.22)
**0.50(0.31,0.81)**	**0.50(0.31,0.82)**	0.62(0.35,1.10)	CAR_PTX	0.85(0.36,1.98)	0.78(0.34,1.82)	1.37(0.52,3.82)	1.33(0.44,4.04)	1.28(0.38,4.56)	0.96(0.23,4.32)	1.4(0.41,4.98)
0.59(0.29,1.18)	0.59(0.29,1.20)	0.73(0.34,1.56)	1.18(0.71,1.94)	CAR_PTX_CET	0.93(0.28,3.07)	1.62(0.44,6.15)	1.57(0.4,6.35)	1.52(0.34,6.96)	1.14(0.21,6.44)	1.66(0.37,7.55)
0.64(0.32,1.26)	0.64(0.33,1.27)	0.79(0.37,1.66)	1.28(0.80,2.05)	1.09(0.55,2.16)	CAR_PTX_MIN	1.75(0.49,6.5)	1.7(0.43,6.93)	1.64(0.37,7.42)	1.23(0.23,6.81)	1.79(0.4,8.09)
**0.37(0.23,0.58)**	**0.37(0.23,0.59)**	**0.45(0.26,0.79)**	0.74(0.52,1.05)	0.63(0.34,1.16)	0.58(0.32,1.04)	CIS	0.97(0.35,2.65)	0.93(0.44,2)	0.7(0.24,2.13)	1.02(0.48,2.17)
**0.38(0.22,0.65)**	**0.38(0.22,0.66)**	**0.46(0.25,0.87)**	0.75(0.47,1.20)	0.64(0.32,1.27)	0.59(0.30,1.14)	1.02(0.66,1.58)	CIS_GEM	0.96(0.27,3.43)	0.72(0.17,3.27)	1.05(0.3,3.79)
**0.39(0.24,0.66)**	**0.40(0.24,0.67)**	**0.49(0.27,0.89)**	0.79(0.52,1.21)	0.67(0.35,1.30)	0.62(0.33,1.17)	1.07(0.85,1.36)	1.05(0.64,1.72)	CIS_IFO	0.75(0.34,1.69)	1.09(0.37,3.18)
**0.53(0.28,0.98)**	0.53(0.28,1.00)	0.65(0.32,1.31)	1.05(0.60,1.84)	0.90(0.42,1.89)	0.82(0.40,1.71)	1.43(0.93,2.20)	1.40(0.76,2.57)	1.33(0.93,1.90)	CIS_IFO_PTX	1.45(0.38,5.49)
**0.36(0.22,0.61)**	**0.36(0.22,0.61)**	**0.45(0.24,0.82)**	0.72(0.47,1.11)	0.61(0.32,1.19)	0.57(0.30,1.07)	0.98(0.77,1.25)	0.96(0.58,1.58)	0.92(0.65,1.28)	0.69(0.42,1.12)	CIS_MIT

The upper-right side shows the comparisons under the Bayesian method (i.e., gray cells), and the lower-left side shows the comparisons under the Frequentist method (i.e., orange cells). Blue cells represent specific interventions, and bolded portions indicate statistically significant results. The result in each cell represents the outcome of the column intervention relative to the row intervention. CIS, cisplatin; MIT, mitolactol; PTX, paclitaxel; GEM, gemcitabine; CAR, carboplatin; BEV, bevacizumab; CET, cetuximab; MIN, nintedanib; ATE, atezolizumab; CAD, cadonilimab.

Furthermore, the ranking of treatment regimens obtained via SUCRA (**Figure 3D**) also indicated that adding pembrolizumab and bevacizumab to the cisplatin plus paclitaxel chemotherapy regimen (87%) was most likely to increase the OS benefit. This was followed by the cadonilimab plus cisplatin and paclitaxel regimen and the atezolizumab plus chemotherapy and bevacizumab regimen (84% vs 83%). Other competitive regimens included cadonilimab plus cisplatin and paclitaxel and bevacizumab (73%), and pembrolizumab plus cisplatin and paclitaxel (71%). It is worth mentioning that the treatment regimen rankings were nearly consistent between the Frequentist (**Figure 3B**) and Bayesian methods(**Figure 3D**), suggesting strong robustness of the results.

### 3.3 Network meta-analysis of PFS

A separate NMA was conducted for PFS, including 14 studies (9–15, 17–23). Isolated treatment regimen nodes (including regimens such as atezolizumab) were removed due to lack of key data, ultimately covering 16 treatment regimens.

**Figure 4:**
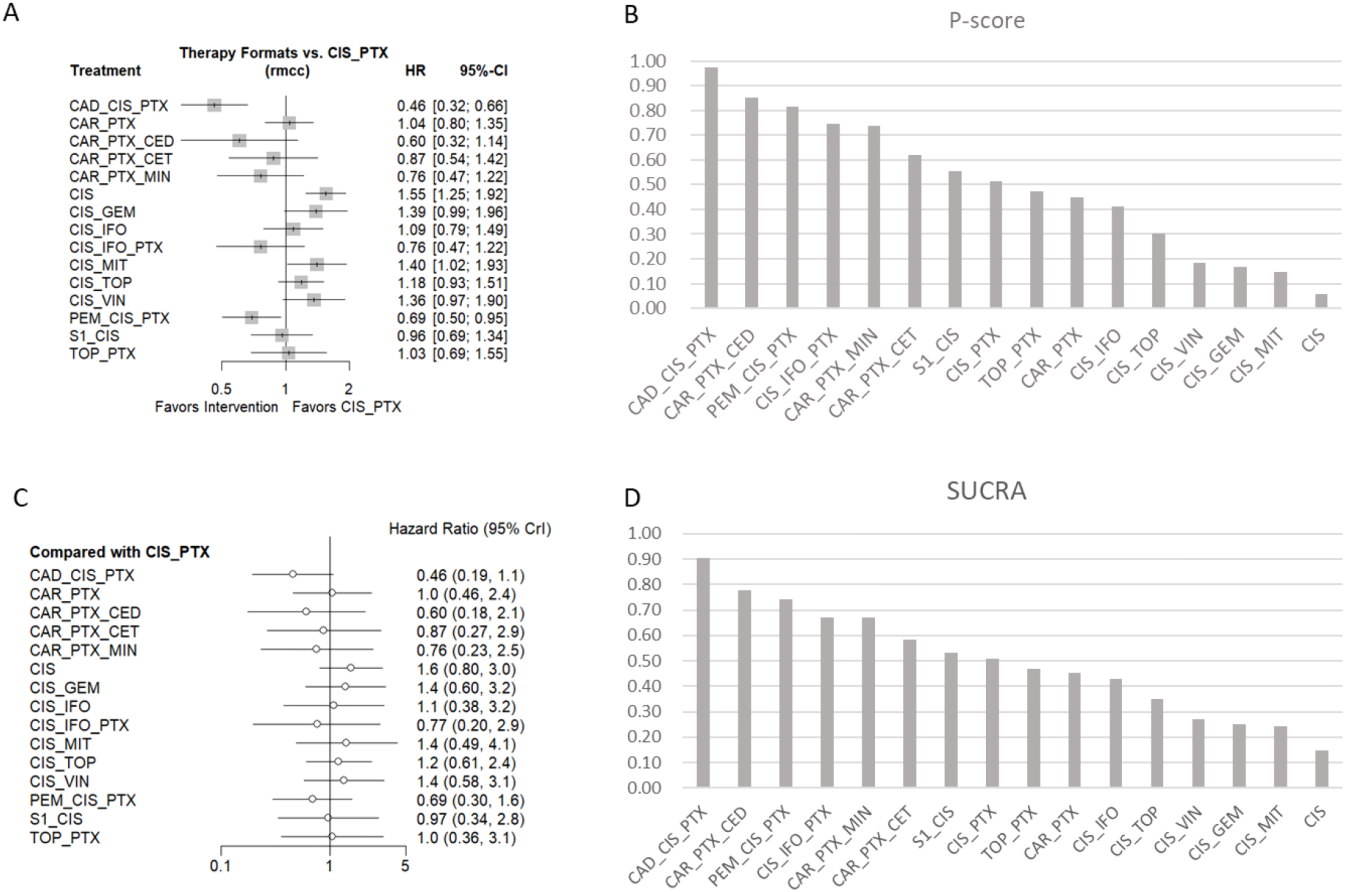
PFS comparisons and ranking based on statistical methods. **(A, B)** show the comparison of different treatment regimens versus the cisplatin plus paclitaxel regimen for PFS, and the probability ranking under the Frequentist method, respectively. **(C, D)** show the comparison of different treatment regimens versus the cisplatin plus paclitaxel regimen for PFS, and the SUCRA ranking under the Bayesian method, respectively. CIS, cisplatin; MIT, mitolactol; PTX, paclitaxel; TOP, topotecan; VIN, vinorelbine; GEM, gemcitabine; CAR, carboplatin; CED, cediranib; S1, teysuno; CET, cetuximab; MIN, nintedanib; CAD, cadonilimab; PEM, pembrolizumab.

The results, as shown in **Figure 4A**, indicated that the cadonilimab plus chemotherapy regimen demonstrated a significant benefit in PFS compared to cisplatin plus paclitaxel (HR: 0.46, 95%CI: 0.32–0.66), ranking first by SUCRA (**Figure 4D**)with a score of 90%. This was followed by the regimen of carboplatin plus paclitaxel combined with cediranib, which showed a benefit compared to cisplatin plus paclitaxel but was not statistically significant (HR: 0.60, 95%CI: 0.32–1.14), with a SUCRA score of 78%. Ranking third was the pembrolizumab plus cisplatin and paclitaxel regimen, which demonstrated a significant benefit compared to cisplatin plus paclitaxel (HR: 0.69, 95%CI: 0.50–0.95), with a SUCRA score of 74%. The pairwise comparisons between all interventions can be found in the **Supplementary Material**. Similarly, under the Bayesian framework, none of the treatment regimens showed a statistically significant advantage compared with the cisplatin plus paclitaxel regimen (**Figure 4C**), whereas under the Frequentist method, some regimens demonstrated statistically significant benefits; nevertheless, the treatment rankings were nearly identical between the two approaches (**Figures 4B, D**).

### 3.4 Summary of safety

Safety Profiles Overall, ICI-based combinations exhibited higher toxicity compared to chemotherapy alone. As summarized in **Table 3**, the incidence of Grade≥3 treatment-related AEs was highest in the quadruple/triple regimens (ICI + chemotherapy + bevacizumab), ranging from 74% to 85%. Notably, the discontinuation rate for any treatment component in these groups reached up to 40.8% (KEYNOTE-826). In contrast, ICI plus chemotherapy without bevacizumab showed a more manageable safety profile (Grade≥3 AEs: 60.4%). Immune-related AEs (irAEs) were predominantly low-grade, with hypothyroidism being the most frequent (11.7%–33.0%). Bevacizumab-containing arms were characterized by distinct toxicities, including hypertension and proteinuria. Traditional chemotherapy backbones primarily resulted in hematological toxicities, consistent with historical data.

**Table 3:** Structured summary of safety profiles across key included trials.

Regimen	Trial	Any grade AEs (%)	Grade ≥3 treatment-emergent AEs (%)	Discontinuation rate (%)	Grade ≥3 irAEs(%)	Common irAEs/AEs
PEM_CIS/CAR_PTX_BEV	KEYNOTE-826	100%	74%	40.80%	16.30%	Hypothyroidism (23.5%), Hyperthyroidism (9.7%), Severe skin reactions (5.1%), Colitis (4.6%)
CIS/CAR_PTX_BEV	KEYNOTE-826	100%	66.80%	32.60%	5.70%	Hypothyroidism (12.4%), Hyperthyroidism (3.6%), Colitis (1%)
PEM_CIS/CAR_PTX	KEYNOTE-826	98.20%	60.40%	19.80%	9.90%	Hypothyroidism (11.7%), Hyperthyroidism (6.3%), Colitis (6.3%)
CIS/CAR_PTX	KEYNOTE-826	98.30%	62.10%	12.10%	4.30%	Hypothyroidism (6%), Hyperthyroidism (2.6%), Colitis (2.6%)
CAD_CIS/CAR_PTX ± BEV	COMPASSION-16	>99%	85%	28%	10%	Hypothyroidism (33%), ALT/AST increased (26%/26%), Rash (25%), Proteinuria (26%)
CIS/CAR_PTX ± BEV	COMPASSION-16	100%	80%	11%	<1%	Hypothyroidism (12%), ALT/AST increased (19%/17%), Rash (7%), Proteinuria (21%)
ATE_CIS/CAR_PTX_BEV	BEATcc	NR	79%	15%	NR	Grade 1–2 hypothyroidism (8%), Grade 1–2 hyperthyroidism (3%)
CIS/CAR_PTX_BEV	BEATcc	NR	75%	16%	NR	Neutropenia (18%), Hypertension (16%)
CIS_PTX	JCOG0505	NR	NR	11.80%	NR	Grade 3 to 4 Neutropenia (85%), Anemia (31.2%), Febrile neutropenia (16.0%)
CAR_PTX	JCOG0505	NR	NR	9.50%	NR	Grade 3 to 4 Neutropenia (76.2%), Anemia (44.4%), Thrombocytopenia (24.6%)

AEs, adverse events; irAEs, immune-related adverse events; NR, not reported; CIS, cisplatin; PTX, paclitaxel; CAR, carboplatin; CED, cediranib; BEV, bevacizumab; ATE, atezolizumab; CAD, cadonilimab; PEM, pembrolizumab.

### 3.5 Heterogeneity, consistency, and sensitivity analyses of the NMA

**Figure 5:**
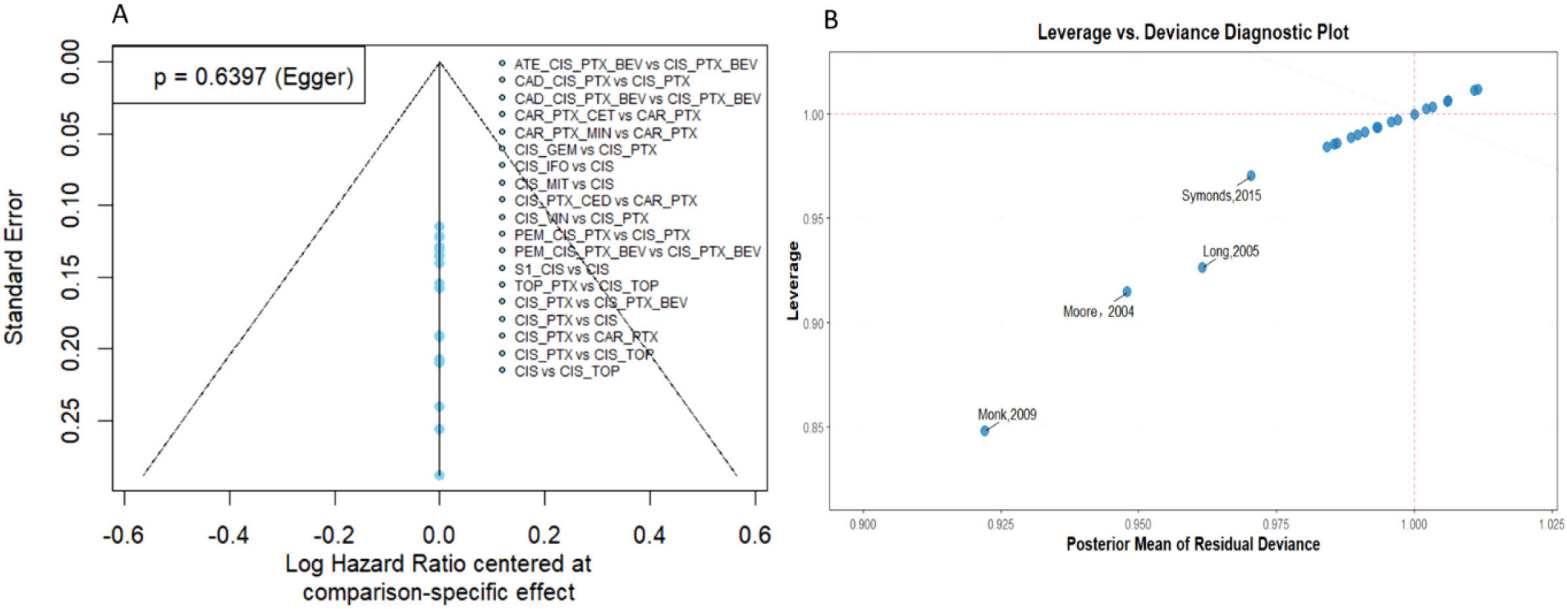
Comparison-adjusted funnel plots **(A)** and deviance-leverage plot **(B)**.

The heterogeneity of the NMA was low across both frameworks. Under the Bayesian random-effects model, the posterior estimate of the between-study standard deviation was  τ=0.256 (95% CrI: 0.012–0.661), corresponding to a variance ( ${\text{τ}}^{2}$) of approximately 0.065. The ${\text{I}}^{2}$ statistic ranged between 0% and 5% for both Frequentist and Bayesian methods, suggesting high similarity and minimal heterogeneity among the included RCTs. Convergence of the Bayesian model was confirmed using Gelman–Rubin diagnostics and trace plots, with all potential scale reduction factors (PSRF) close to 1.0, indicating adequate mixing (see **Supplementary Material 1**). Consistency was rigorously assessed through both global and local approaches. The global design-by-treatment interaction test showed that the DIC for the consistency model (41.50) was nearly identical to that of the unrelated mean effects (UME) model (41.95). A ΔDIC < 5 indicates that the consistency model effectively captured the data variability without significant global inconsistency. Furthermore, local node-splitting results (refer to **Supplementary Material 1**) revealed no significant differences between direct and indirect comparisons ( P > 0.05), demonstrating strong coherence across the treatment network. The risk of publication bias was assessed using comparison-adjusted funnel plots (**Figure 5A**). The results indicated no significant publication bias, as evidenced by the symmetrical distribution in the funnel plots and an Egger’s test p-value > 0.05. Sensitivity analyses were conducted to evaluate the robustness of the findings. A prior sensitivity analysis was performed by comparing the primary model with an alternative specification using a half-normal prior ( τ∼HN(0,1)). The results were highly consistent, with a negligible change in DIC (41.68) and stable SUCRA rankings. Cross-validation between the Frequentist and Bayesian approaches showed that while some treatment regimens reached statistical significance under the Frequentist framework, their 95% credible intervals crossed the null under the Bayesian framework. This divergence may be attributed to the Bayesian model’s more conservative integration of the full posterior uncertainty of τ. To investigate potential outliers and their impact on the model, we performed an influence analysis using a deviance-leverage plot (see **Figure 5B**). The contributions to deviance and leverage for all 15 trials clustered near 1, suggesting that the results were robust and not disproportionately driven by any individual study. Meanwhile, the ranking scores and the identification of the most efficacious regimens remained nearly identical between the two methods, confirming the overall robustness of the study's conclusions. Finally, we conducted a subgroup network meta-analysis based on PD-L1 CPS ≥ 1, while removing one study with moderate risk (Mountzios, 2009) and an older study (GOG-110) for sensitivity analysis. The results confirmed that the relative efficacy ranking remained stable (see **Supplementary Material 1**).

## 4 Discussion

This study systematically integrated 15 RCTs, enrolling a total of 4,588 patients with R/MCC. Through NMA within both Bayesian and Frequentist method, we compared the relative efficacy of 21 drug regimens regarding OS and PFS. However, the validity of PFS as a surrogate for OS in cervical cancer treated with ICIs warrants caution. While PFS is a robust indicator of initial treatment response, its correlation with OS can be confounded by subsequent lines of therapy and the unique 'delayed' survival benefit characteristic of immunotherapy (25, 26). The results indicate that ICI combination regimens (with or without bevacizumab) show a significant advantage in OS, while cisplatin plus paclitaxel remains the most efficacious basic chemotherapy regimen. To the best of our knowledge, this is a key NMA to incorporate immunotherapy regimens for R/MCC, which fills the evidence gap caused by the lack of direct head-to-head trials in this setting and provides more comprehensive indirect comparison evidence for clinical strategy selection.

Firstly, this study confirms that multiple ICI combination regimens are significantly superior to traditional chemotherapy in terms of OS and PFS. The SUCRA results show that pembrolizumab combined with chemotherapy, optionally with bevacizumab, has the greatest potential for OS benefit, followed by cadonilimab combined with chemotherapy ± bevacizumab and atezolizumab combined with chemotherapy and bevacizumab. These findings are highly consistent with the key conclusions of recent large-scale clinical trials. The KEYNOTE-826 study demonstrated that pembrolizumab combined with chemotherapy ± bevacizumab achieved a mOS of 28.6 months in patients with PD-L1-positive tumors (CPS ≥1). Pembrolizumab improved OS rates regardless of bevacizumab use (23). Although the incidence of Grade 3 or higher adverse events was higher in the pembrolizumab group compared to the placebo group (82.4% vs. 75.4%), no negative impact on quality of life was observed (27).The COMPASSION-16 study, based on a Chinese population, confirmed the survival benefits of the bispecific antibody cadonilimab (22). These benefits were independent of bevacizumab co-administration or PD-L1 expression status. In this study, the incidence of Grade 3 or higher adverse events was 82% in the treatment group versus 79% in the control group. Furthermore, the BEATcc trial showed that adding atezolizumab to standard treatment significantly improved PFS and OS, establishing a new first-line treatment option for advanced cervical cancer (21). However, as all patients in this trial received bevacizumab, the benefits for patients ineligible for bevacizumab remain unclear. The incidence of Grade 3 or higher adverse events in the BEATcc trial was 79%, compared to 75.4% in the control group. While our SUCRA rankings identify ICI plus chemotherapy combined with bevacizumab as the most efficacious regimen, the high incidence of grade≥3 AEs and treatment discontinuation rates must be carefully considered. Clinicians should balance maximal therapeutic efficacy with individual patient fitness, particularly assessing contraindications to bevacizumab such as risk of fistula or bleeding.

Secondly, this study reaffirms that the cisplatin or carboplatin plus paclitaxel regimen remains the backbone chemotherapy, superior to other chemotherapy regimens such as ifosfamide plus cisplatin or topotecan plus paclitaxel. This result is consistent with previous research. Since the publication of the GOG-26 Phase II trial, single-agent cisplatin has been considered the standard chemotherapy for cervical cancer (28). Building upon this, Gynecologic Oncology Group(GOG) conducted a series of explorations on combination regimens: GOG-110 compared ifosfamide plus cisplatin (10), GOG-169 compared paclitaxel plus cisplatin (11), and GOG-179 compared topotecan plus cisplatin (12), all using single-agent cisplatin as the control. Combined with the results of GOG-204 (13), the cisplatin plus paclitaxel regimen emerged as superior. Notably, the JCOG-0505 study showed that the carboplatin plus paclitaxel regimen was non-inferior to cisplatin plus paclitaxel in terms of PFS and OS, with significantly reduced toxicity (9). A *post-hoc* analysis suggested an advantage for carboplatin plus paclitaxel in patients who had not previously received cisplatin. The AGO-Zervix-1 study, which compared non-platinum regimens with platinum regimens, also supports platinum-based combination chemotherapy as the standard first-line chemotherapy for R/MCC patients (20, 29).

However, the effective duration of response to platinum-based chemotherapy is short. Adding anti-angiogenic agents or ICIs can effectively increase patient benefit, a fact also confirmed by this study. The combination of bevacizumab and immune checkpoint inhibitors (ICIs) exerts a synergistic immunomodulatory effect, enhancing the efficacy of ICIs by augmenting antigen presentation, activating cytotoxic CD8^+^T cells, and promoting lymphocyte infiltration into the tumor microenvironment (30). The GOG-227C study demonstrated its efficacy in 46 pre-treated R/MCC patients, with PFS and OS of 3.4 months and 7.3 months, respectively (31). Consequently, the FDA approved bevacizumab for refractory R/MCC treatment. It is worth noting that other anti-angiogenic agents, such as pazopanib, cediranib, lapatinib, and nintedanib, showed limited activity and potentially increased the incidence of adverse events (15, 19, 32–34), while a Phase II study of cetuximab in R/MCC also did not show significant clinical benefit (35).

In the field of immunotherapy, besides the three ICIs included in this study (pembrolizumab, cadonilimab, and atezolizumab), nivolumab, cemiplimab, zimberelimab, socazolimab, enlonstobart, and tuvonralimab/iparomlimab have also been approved by China's National Medical Products Administration (NMPA) for second-line or later R/MCC treatment. Currently, Phase III clinical trials DUBHE-C-204 (tuvonralimab/iparomlimab combination antibody + cisplatin or carboplatin plus paclitaxel ± bevacizumab) and VICT-004 (zimberelimab + platinum-containing chemotherapy ± bevacizumab) are ongoing, promising to offer more appropriate and cost-effective treatment options for R/MCC patients. Furthermore, research is actively exploring chemotherapy-free combination therapy modes, such as targeted therapy combined with immunotherapy. For instance, the ALTER-GO-020 study explored penpulimab plus anlotinib as first-line therapy for R/MCC, showing a high Objective Response Rate (ORR) of 58.8% in preliminary results, but limitations exist due to the small sample size (36). The SHR-1210-II-217 Phase II study compared camrelizumab plus famitinib, camrelizumab monotherapy, and chemotherapy alone, with ORRs of 42.9%, 22.2%, and 14.3%, and median OS of 20.6 months, 14.9 months, and 13.9 months, respectively. This demonstrates a clear benefit from the combined targeted and immune approach, especially in patients with squamous cell carcinoma or PD-L1 positivity (37). A Phase III trial of this regimen (SHR1210-III-329) is ongoing.

Finally, with the advent of antibody-drug conjugates (ADCs), the therapeutic landscape for patients with R/MCC has further expanded. The Phase III innovaTV 301 trial compared single-agent Tisotumab vedotin with investigator's choice chemotherapy (including topotecan, vinorelbine, gemcitabine, irinotecan, and pemetrexed) in 502 R/MCC patients who progressed during or after first-line treatment. The results showed a survival benefit for Tisotumab vedotin over chemotherapy (38), leading to FDA approval for R/MCC treatment. As more drug trials are conducted and initiated, they are expected to bring further benefits to patients, particularly in reducing associated drug toxicities and improving patient quality of life. For example, bevacizumab use may cause hypertension, proteinuria, or hemorrhage, while immunotherapy may trigger systemic autoimmune reactions in various organs, including thyroid dysfunction and dermatological lesions.

## 5 Limitations

While this study provides valuable indirect evidence comparing pharmacological treatments for R/MCC, several limitations must be acknowledged:

Firstly, the indirect nature and inherent heterogeneity of the data sources. Although our analysis was restricted to RCTs, the majority of the comparisons were based on indirect evidence. Heterogeneity may exist across the included studies regarding patient characteristics (e.g., the proportion of squamous cell carcinoma vs. non-squamous cell carcinoma), trial designs, and follow-up durations. Despite favorable consistency results—with Deviance Information Criterion (DIC) differences of less than 5 and node-splitting P-values greater than 0.05—potential residual heterogeneity may still impact the accuracy of the network effect size estimates.

Secondly, the discrepancies between Bayesian and Frequentist results. In the analysis of OS and PFS, comparisons under the Bayesian framework tended to be less statistically significant than those using Frequentist methods. While the Bayesian approach provides a more comprehensive quantification of uncertainty (resulting in wider 95% Credible Intervals), this lack of significance may affect clinical confidence in the efficacy results. Our sensitivity analysis and deviance-leverage plot indicate robust results.

Thirdly, the potential loss of information due to node merging or exclusion. Specifically, we simplified "cisplatin or carboplatin plus paclitaxel" into a single "cisplatin plus paclitaxel" node. Although clinical evidence supports the non-inferiority of carboplatin to cisplatin, this consolidation may mask subtle differences in toxicity and efficacy between different platinum agents within specific subgroups. Meanwhile, some nodes were excluded in the network of PFS, which has influence on the rank of PFS. Furthermore, a key challenge in immuno-oncology trials is the presence of non-proportional hazards (NPH), such as late separation of survival curves. In such cases, the constant HR may not fully capture the dynamic benefit of the treatment (39). Future research could benefit from employing alternative estimands like Restricted Mean Survival Time (RMST). RMST measures the life expectancy within a specific time window and does not rely on the PH assumption, offering a more nuanced interpretation of survival gains when curves converge or cross (40, 41).

Finally, the lack of comprehensive safety and toxicity data. Due to inconsistencies in the reporting methods and definitions of adverse events (such as hypertension, hemorrhage, or autoimmune reactions) across the various trials, we were unable to include these metrics in the network meta-analysis. This remains a critical gap, as safety profiles are paramount for clinical decision-making. To address these limitations, the Confidence-in-NMA (CINeMA) framework could be employed in future studies to rigorously evaluate the certainty of evidence in network meta-analysis results (42).

## Data Availability

The original contributions presented in the study are included in the article/**Supplementary Material**. Further inquiries can be directed to the corresponding author.
