# Supplementary material for: Comparison of drug regimens for recurrent or metastatic cervical cancer: a systematic review and network meta-analysis
Source: Front Immunol. 2026 Feb 25;17:1775409. doi: 10.3389/fimmu.2026.1775409 (PMC12975740; doi:10.3389/fimmu.2026.1775409)

**Catalog**

[**1.** **The results retrieved from different databases** 2](#_Toc221420334)

[**2.** **Risk of bias assessment** 3](#_Toc221420335)

[**3.** **Gelman–Rubin diagnostics and trace plots of OS** 4](#_Toc221420336)

[**4.** **Gelman–Rubin diagnostics and trace plots of PFS** 6](#_Toc221420337)

[**5.** **Characteristics of included studies** 7](#_Toc221420338)

[**6.** **Node-splitting results** 9](#_Toc221420339)

[**7.** **Summary of sensitivity analysis results（OS）** 9](#_Toc221420340)

[**8.** **HRs and SUCRA rankings after excluding GOG-110 and Mountzios (2009)** 10](#_Toc221420341)

1. **The results retrieved from different databases**

| Database | Search strategy | Result |
| --- | --- | --- |
| Pubmed | ("Uterine Cervical Neoplasms"[Mesh] OR "Cervical Cancer" OR "Cervical Carcinoma") AND (Recurrent OR Metastatic OR Advanced OR Relapsed) AND ("Drug Therapy"[Mesh] OR Chemotherapy OR Immunotherapy OR "Molecular Targeted Therapy" OR "Immune Checkpoint Inhibitors") AND ("Randomized Controlled Trial" OR "Clinical Trial") | 1287 |
| Web of Science | TS = ("Uterine Cervical Neoplasms" OR "Cervical Cancer" OR "Cervical Carcinoma") AND TS=(Recurrent OR Metastatic OR Advanced OR Relapsed) AND TS=("Drug Therapy" OR Chemotherapy OR Immunotherapy OR "Molecular Targeted Therapy" OR "Immune Checkpoint Inhibitors") AND TS=("Randomized Controlled Trial" OR "Clinical Trial") | 748 |
| Cochrane Central Register of Controlled Trials | ("Uterine Cervical Neoplasms" OR "Cervical Cancer" OR "Cervical Carcinoma") AND (Recurrent OR Metastatic OR Advanced OR Relapsed) AND ("Drug Therapy" OR Chemotherapy OR Immunotherapy OR "Molecular Targeted Therapy" OR "Immune Checkpoint Inhibitors") | 1376 |
| Embase | ('uterine cervical neoplasms'/exp OR 'uterine cervical neoplasms' OR 'cervical cancer'/exp OR 'cervical cancer' OR 'cervical carcinoma'/exp OR 'cervical carcinoma') AND ('recurrent' OR 'metastatic' OR 'advanced' OR 'relapsed') AND ('drug therapy'/exp OR 'drug therapy' OR 'chemotherapy'/exp OR 'chemotherapy' OR 'immunotherapy'/exp OR 'immunotherapy' OR 'molecular targeted therapy'/exp OR 'molecular targeted therapy' OR 'immune checkpoint inhibitors'/exp OR 'immune checkpoint inhibitors') AND 'clinical trial'/lnk AND [<1966-2026]/py | 1965 |
| Total | - | 5376 |

1. **
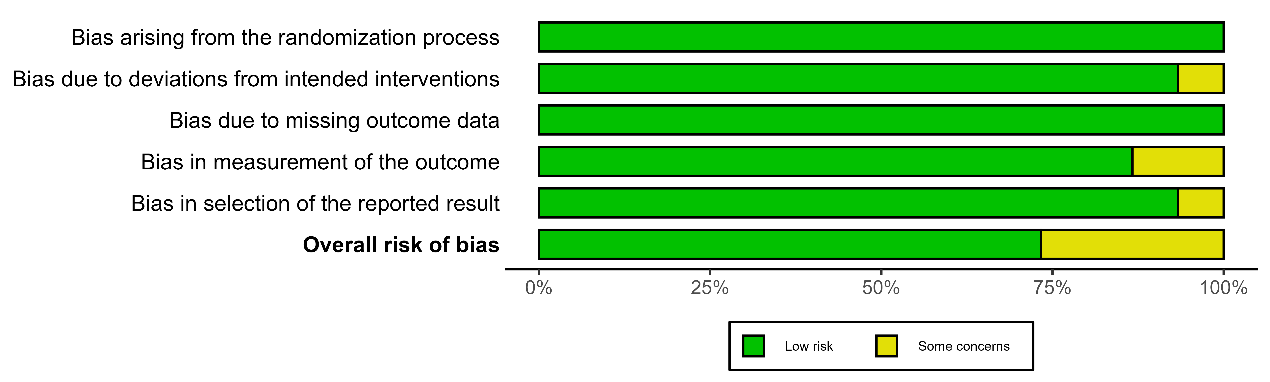
Risk of bias assessment**


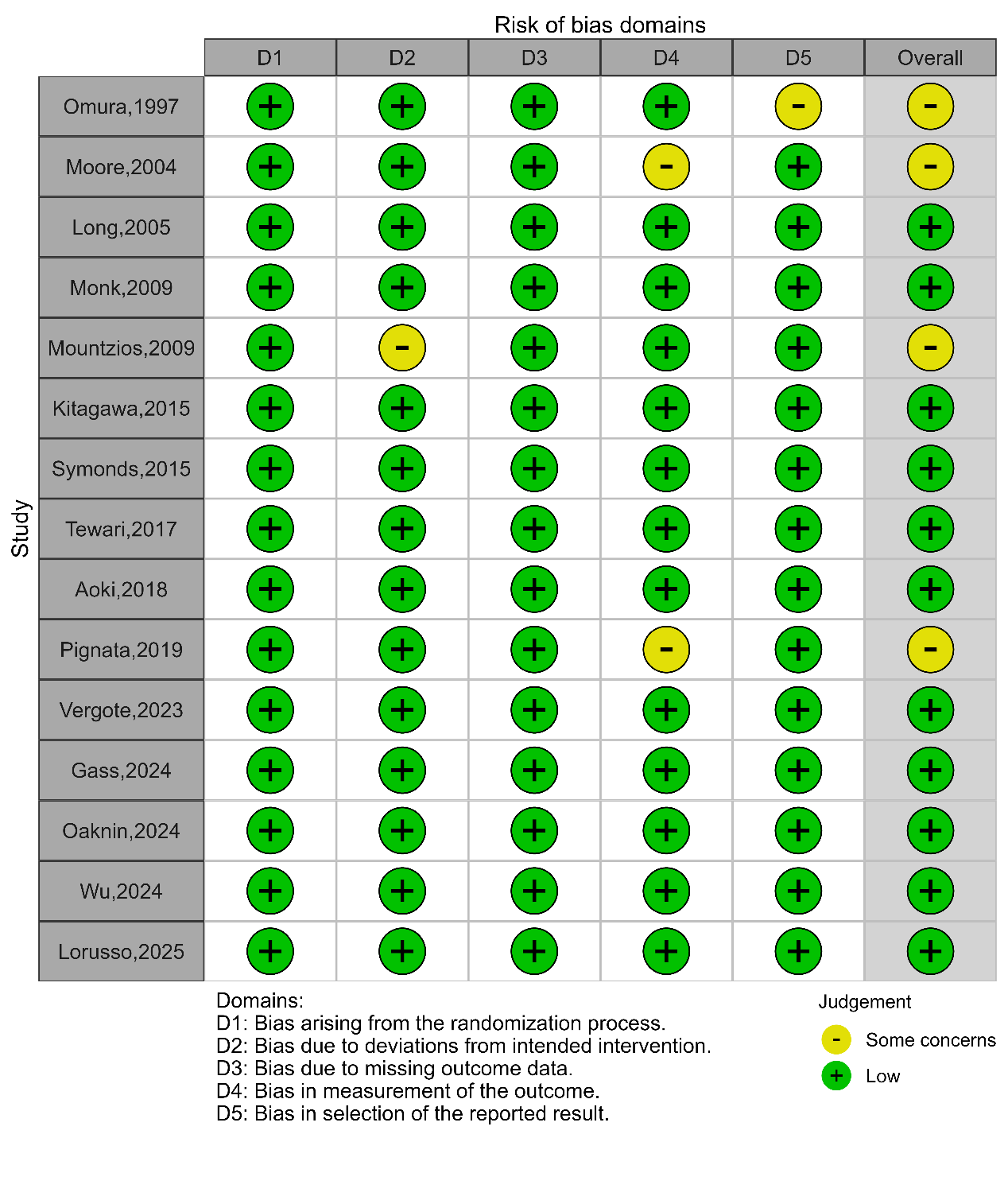


1. **Gelman–Rubin diagnostics and trace plots of OS**


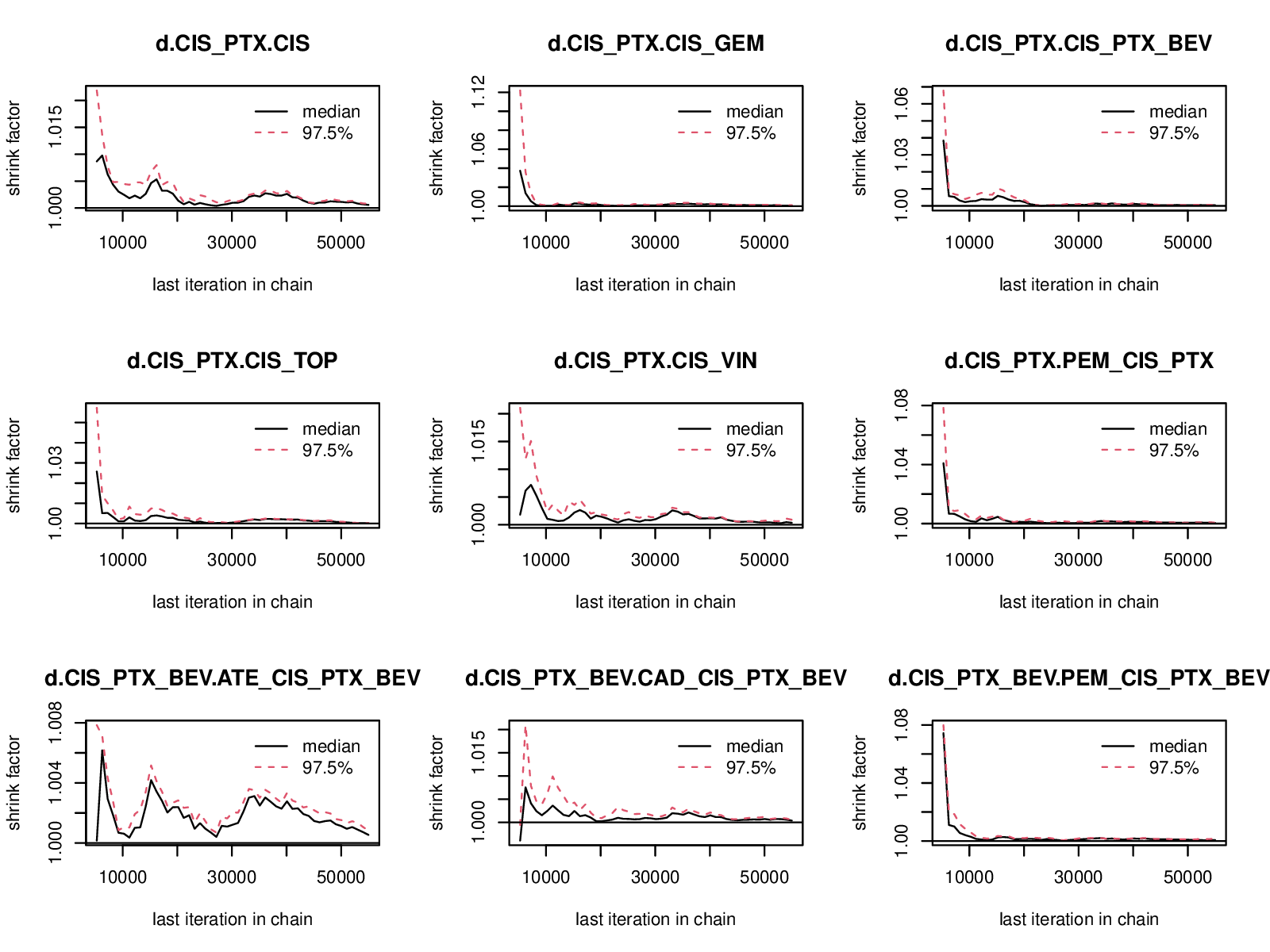

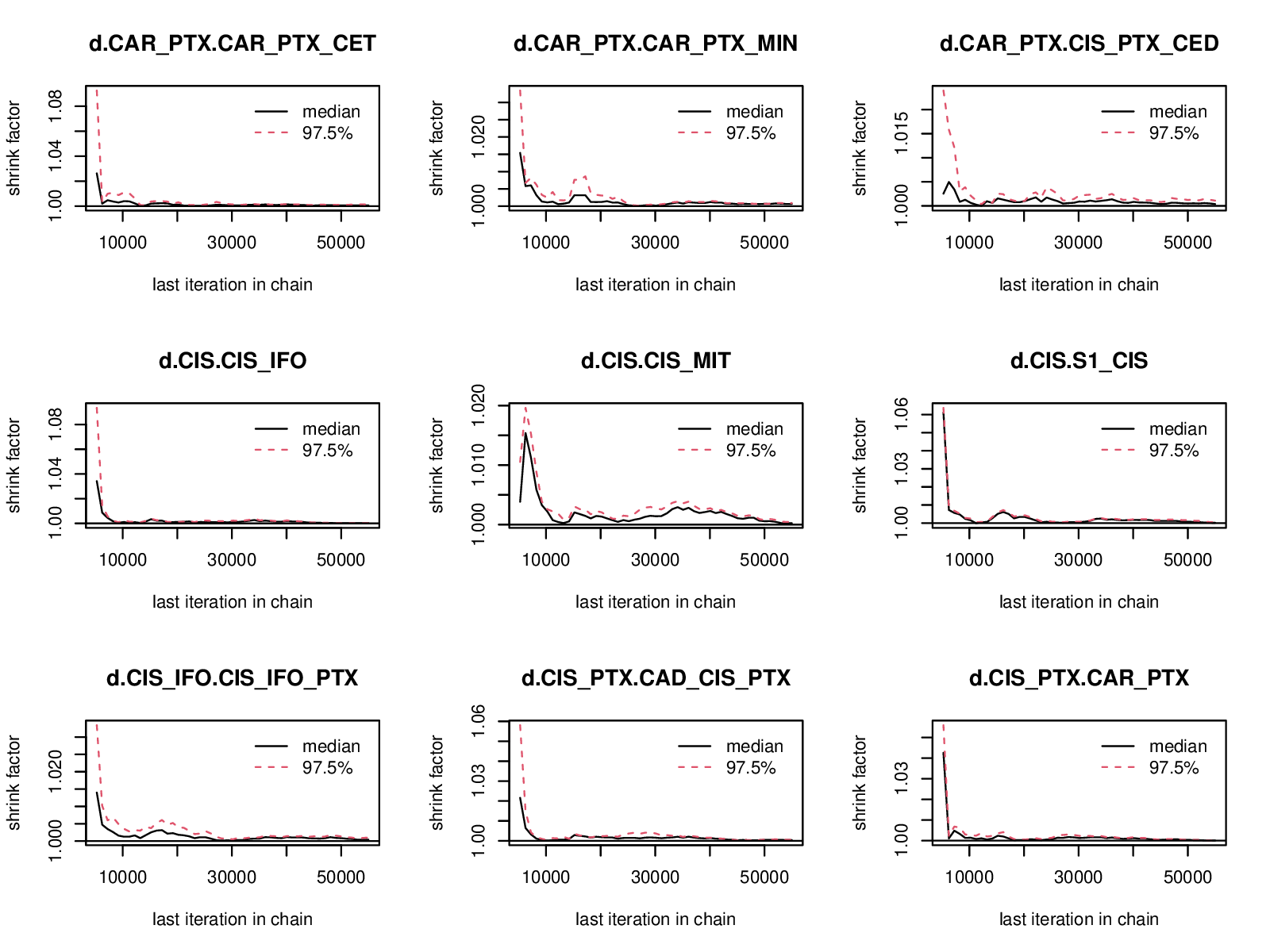


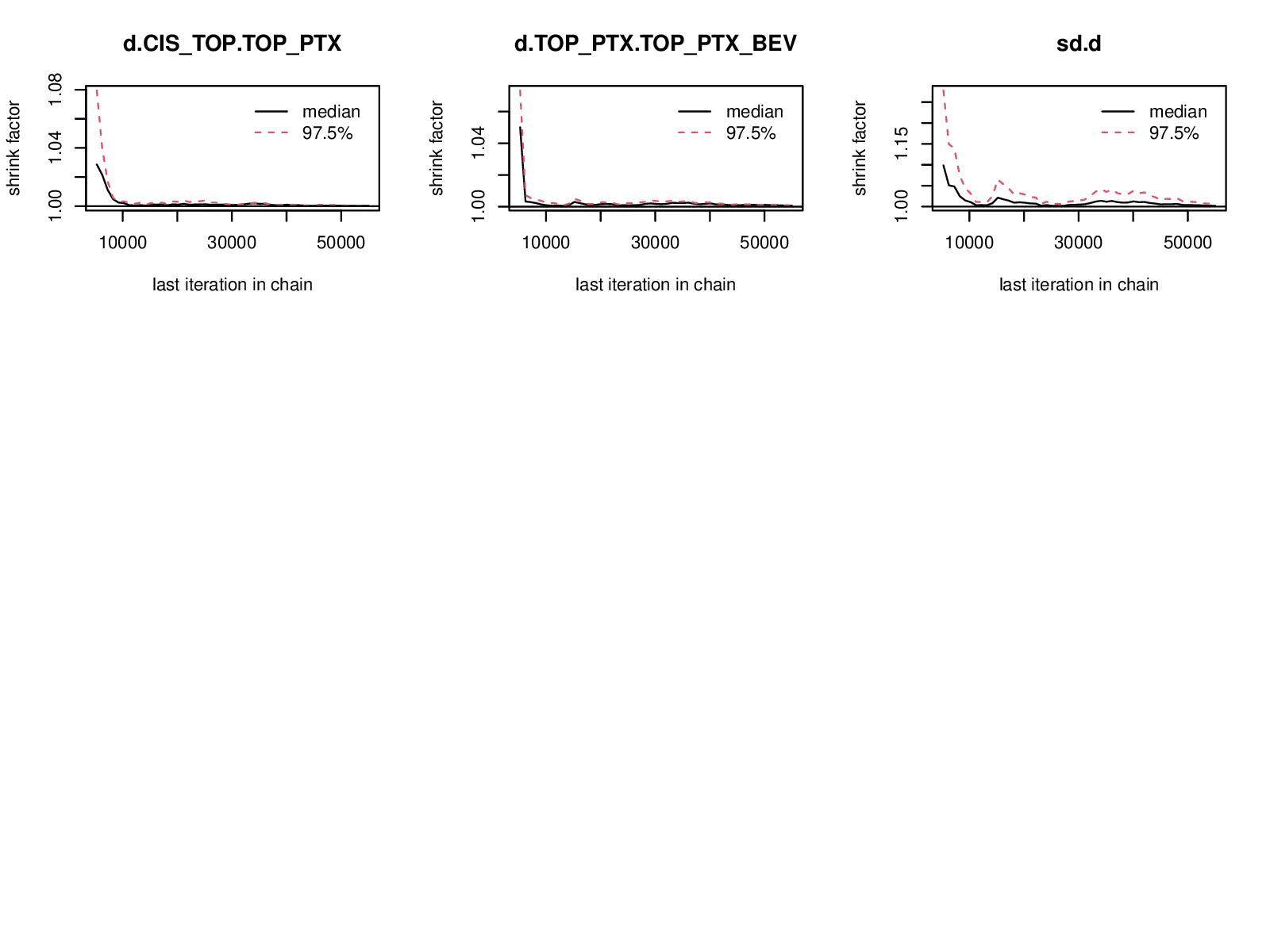


1. **Gelman–Rubin diagnostics and trace plots of PFS**


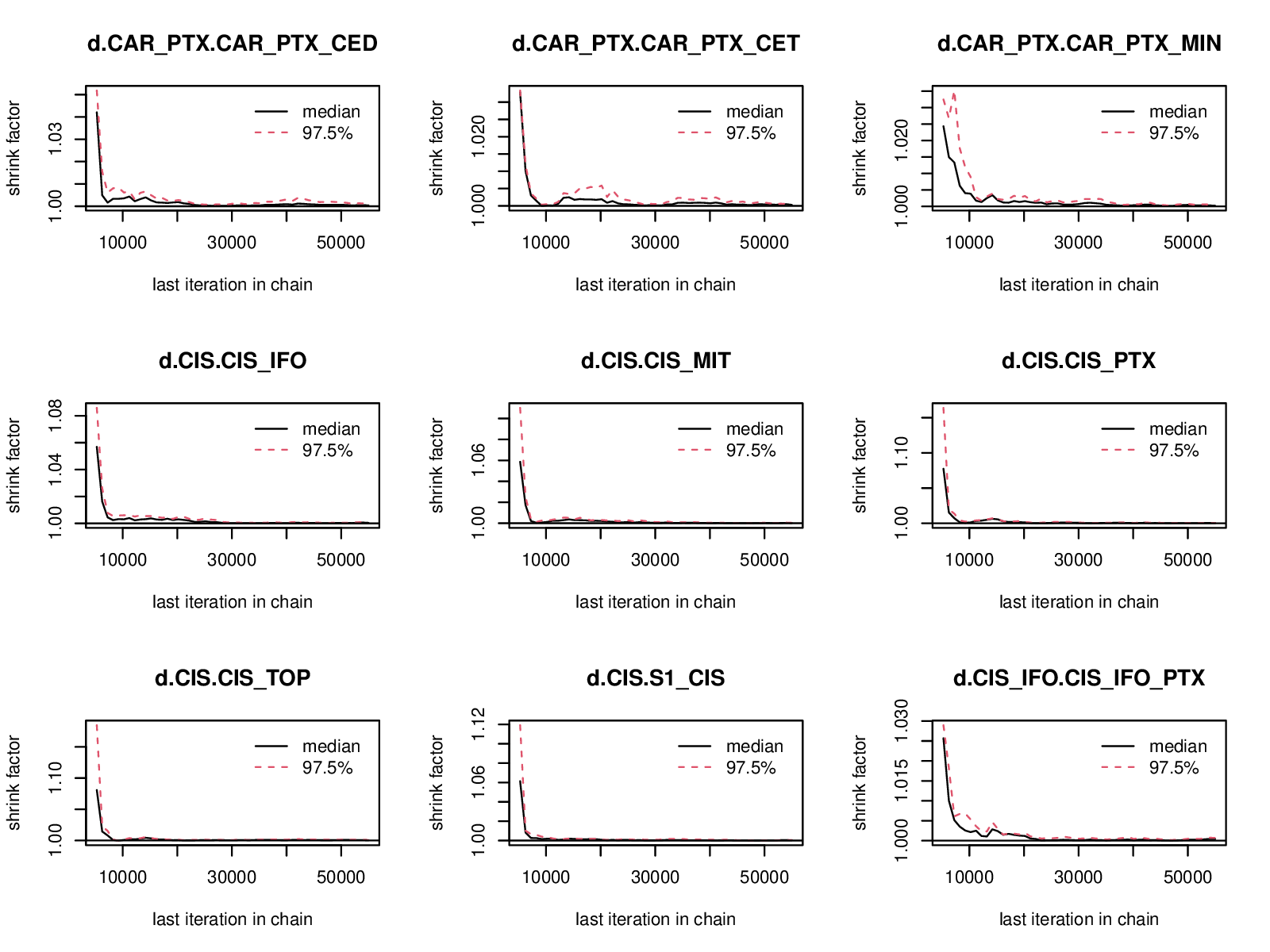

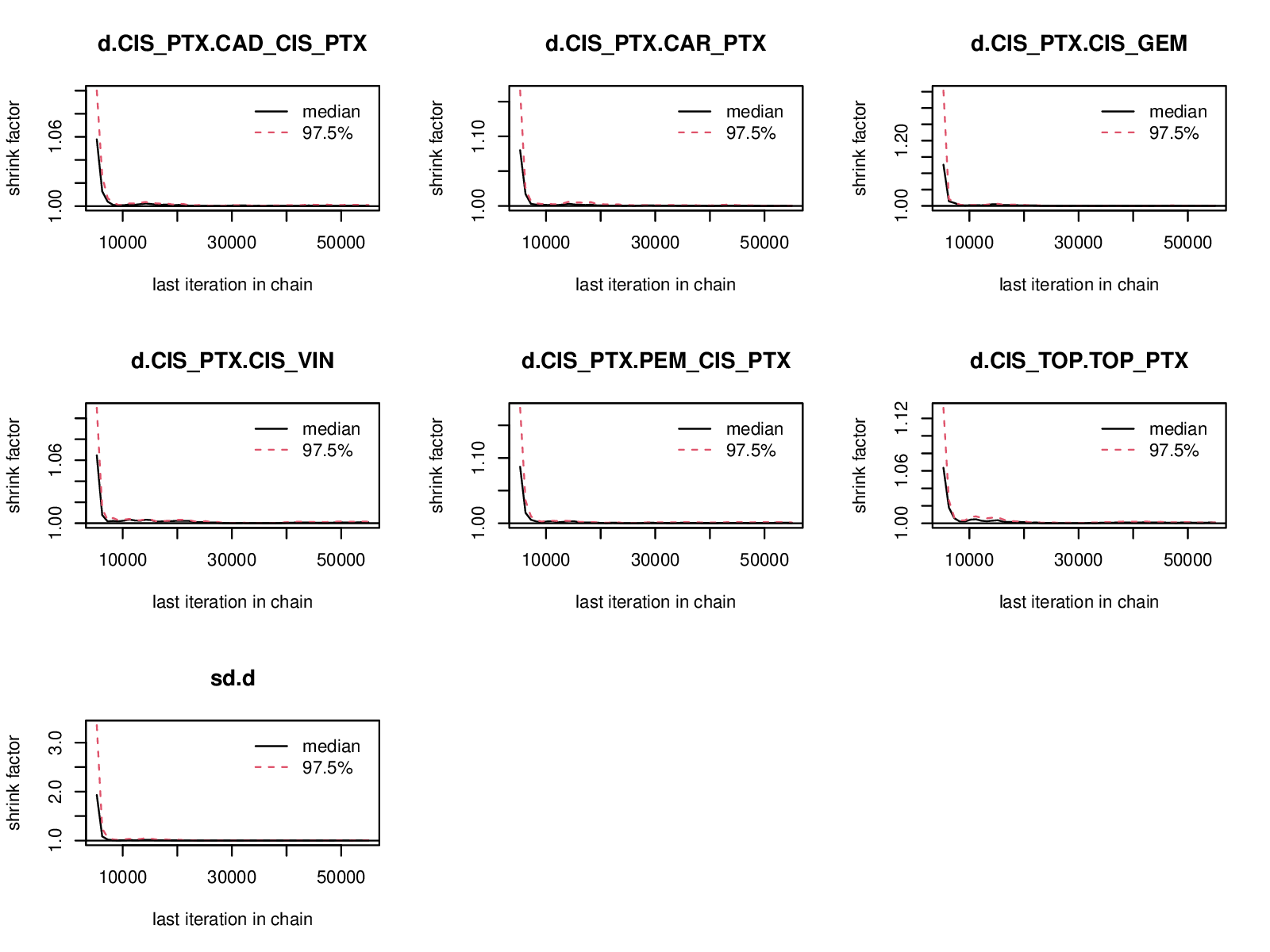


1. **Characteristics of included studies**

| Trial name / Registration ID | Treatment arm | Sample size | Performance status 0-1 (%) | Squamous cell carcinoma (%) | PD-L1 CPS ≥1 (%) | Prior platinum exposure, including radiosensitizing (%) | The use of BEV | geographic Region |
| --- | --- | --- | --- | --- | --- | --- | --- | --- |
| GOG-110 / NA | CIS | 140 | 85.7% | 100% | NR | 13.6% | NO | NR |
|  | CIS_MIT | 147 | 85.4% | 100% | NR | 16.3% | NO |  |
|  | CIS_IFO | 151 | 85.5% | 100% | NR | 13.9% | NO |  |
| GOG-169 / NA | CIS | 134 | 91.8% | 100% | NR | 29.8% | NO | NR |
|  | CIS_PTX | 130 | 86.9% | 100% | NR | 23.8% | NO |  |
| GOG-179 / NA | CIS | 146 | 92% | 83% | NR | 56% | NO | NR |
|  | CIS_TOP | 147 | 92% | 87% | NR | 58% | NO |  |
| GOG-204 / NA | CIS_PTX | 103 | 100% | 78.6% | NR | 68.0% | NO | NR |
|  | CIS_VIN | 108 | 100% | 74.1% | NR | 73.1% | NO |  |
|  | CIS_GEM | 112 | 100% | 78.6% | NR | 64.3% | NO |  |
|  | CIS_TOP | 111 | 100% | 77.5% | NR | 73.0% | NO |  |
| Mountzios,2009, (14) | CIS_IFO | 73 | 83% | 80% | NR | 35.6% | NO | Greece |
|  | CIS_IFO_PTX | 76 | 88% | 67% | NR | 51.3% | NO |  |
| JCOG-0505/ NCT00295789 | CIS_PTX | 127 | 98% | 83% | NR | 48% | NO | Japan |
|  | CAR_PTX | 126 | 97% | 83% | NR | 57% | NO |  |
| CIRCCa / ISRCTN23516549 | CAR_PTX | 35 | 100% | 74% | NR | 83% | NO | United Kingdom |
|  | CAR_PTX_CED | 34 | 100% | 64% | NR | 82% | NO |  |
| GOG240 / NCT008030 | CIS/TOP_PTX | 225 | 100% | 68% | NR | 74% | NO | Global (USA, Canada, and Spain) |
|  | CIS/TOP_PTX_BEV | 227 | 100% | 70% | NR | 75% | YES |  |
| NA / NCT00770874 | S1_CIS | 188 | 100% | 100% | NR | 64.4% | NO | Global (Japan, Korea, and Taiwan) |
|  | CIS | 174 | 100% | 100% | NR | 63.8% | NO |  |
| MITO CERV-2 / NCT00997009 | CAR_PTX | 52 | 100% | 79% | NR | 58% | NO | Italy |
|  | CAR_PTX_CET | 55 | 100% | 78% | NR | 56% | NO |  |
| BGOG/ENGOT-cx1 / NCT0200 9579 | CAR_PTX_NIN | 62 | NR | 52% | NR | 55% | NO | Global (Belgium, Spain, Italy and Germany) |
|  | CAR_PTX | 58 | NR | 72% | NR | 55% | NO |  |
| AGO-Zervix-1 / NCT01405235 | TOP_PTX | 88 | 92% | NR | NR | NR | NO | Global (Germany, Austria, and Belgium) |
|  | CIS_TOP | 85 | 89.6% | NR | NR | NR | NO |  |
| BEATcc / NCT03556839 | ATE_CIS/CAR_BEV | 206 | 100% | 80% | NR | 65% | YES | Global (Europe, Japan, and the USA) |
|  | CIS/CAR_BEV | 204 | 99% | 77% | NR | 66% | YES |  |
| COMPASSION-16/NCT04982237 | CAD_CIS/CAR_PTX±BEV | 222 | 100% | 82% | 70% | 48% | optional | China |
|  | CIS/CAR_PTX±BEV | 223 | 100% | 84% | 54% | 48% | optional |  |
| KEYNOTE-826/ NCT03635567 | PEM_CIS/CAR_PTX_BEV | 196 | 99.5% | 75% | 89.3% | 56.1% | YES | Global (19 countries) |
|  | CIS/CAR_PTX_BEV | 193 | 100% | 63.2% | 49.2% | 51.8% | YES |  |
|  | PEM_CIS/CAR_PTX | 112 | 99.1% | 78.6% | 49.1% | 57.2% | NO |  |
|  | CIS/CAR_PTX | 116 | 100% | 76.7% | 89.7% | 63.8% | NO |  |
| CIS, cisplatin; MIT, mitolactol; PTX, paclitaxel; TOP, topotecan; VIN, vinorelbine; GEM, gemcitabine; CAR, carboplatin; CED, cediranib; BEV, bevacizumab; S1, teysuno; CET, cetuximab; MIN, nintedanib; ATE, atezolizumab; CAD, cadonilimab; PEM, pembrolizumab; NA, not available; NR, not reported. | | | | | | | | |

1. **Node-splitting results**


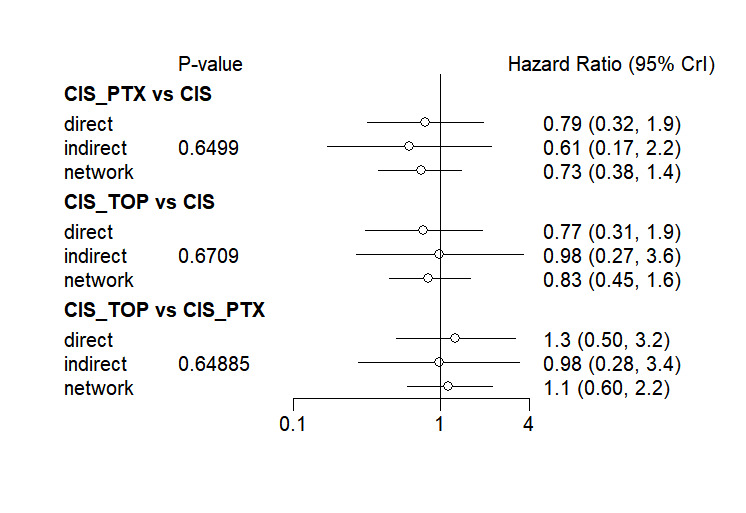


1. **Summary of sensitivity analysis results（OS）**

| **Comparison Dimension** | **Number of Studies (K)** | **Best Regimen (Rank 1)** | **Heterogeneity (τ2)** | **Consistency of Conclusion** |
| --- | --- | --- | --- | --- |
| Primary Analysis | 15 | PEM_CIS_PTX_BEV | 0.065 | - |
| Subgroup Analysis: PD-L1 CPS ≥ 1 | 3 | PEM_CIS_PTX_BEV | 0.064 | Consistent |
| Omitting two study: GOG-110 and Mountzios,2009 | 12 | PEM_CIS_PTX_BEV | 0.058 | Consistent |

1. **HRs and SUCRA rankings after excluding GOG-110 and Mountzios (2009)**


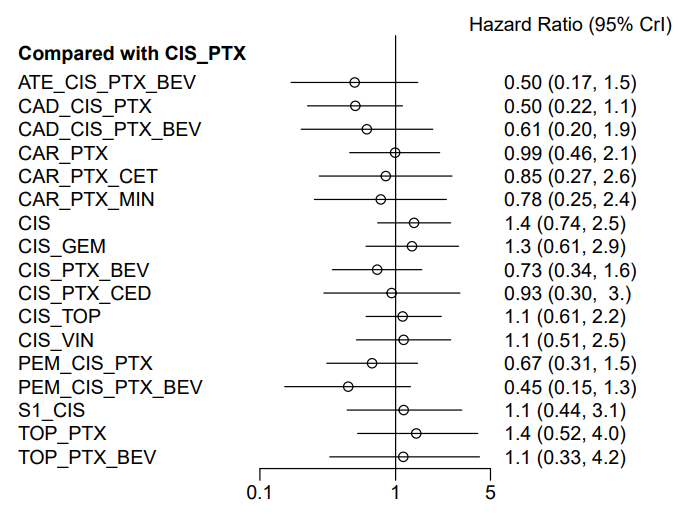

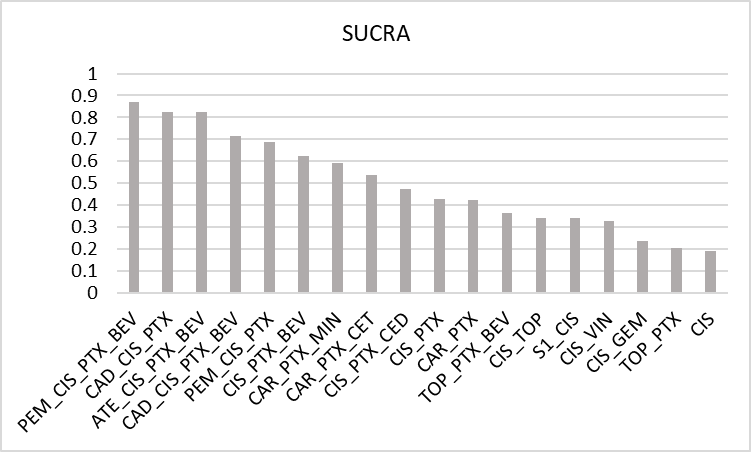

Supplement: Supplementary file 1 [file Table1.docx]
